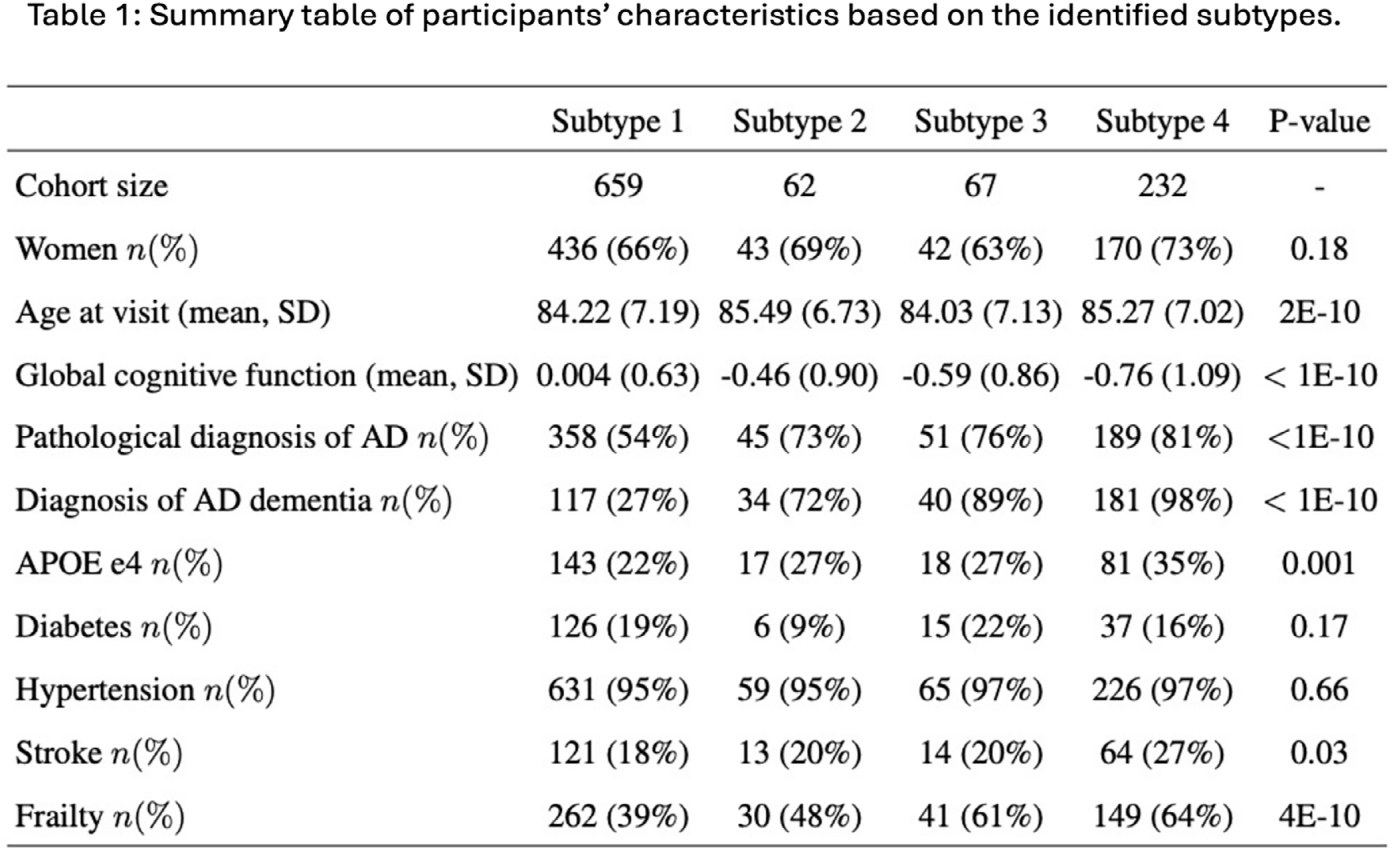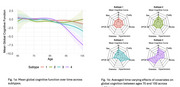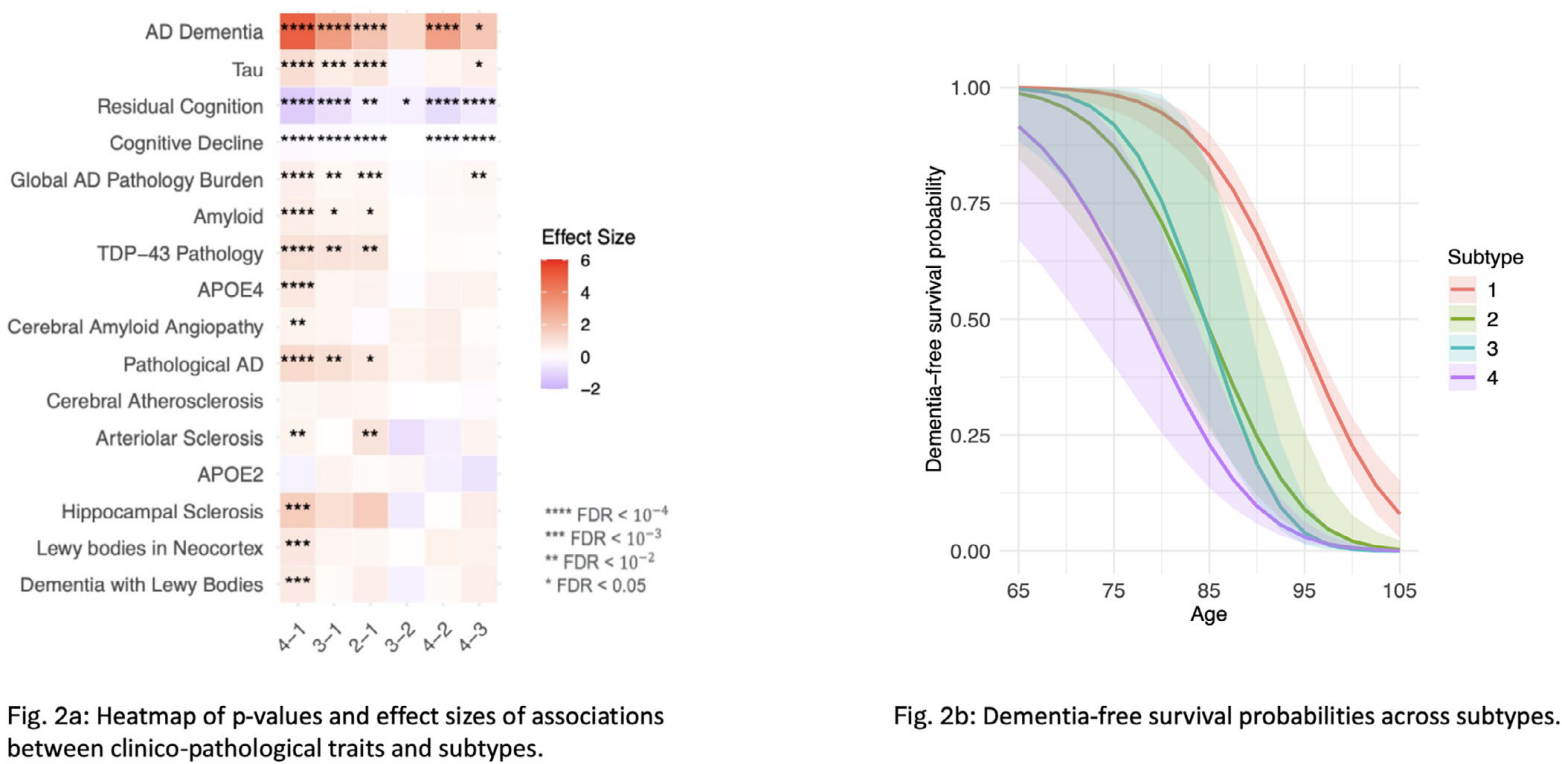# TPClust: Temporal Profile‐Guided Disease Subtyping Using High‐Dimensional Omics Data

**DOI:** 10.1002/alz70860_103996

**Published:** 2025-12-23

**Authors:** Boyi Hu, Badri N. Vardarajan, Philip L. De Jager, David A. A. Bennett, Yuanjia Wang, Annie J. Lee

**Affiliations:** ^1^ Columbia University, New York, NY, USA; ^2^ Columbia University Irving Medical Center, New York, NY, USA; ^3^ The Gertrude H. Sergievsky Center, College of Physicians and Surgeons, Columbia University, New York, NY, USA; ^4^ Department of Neurology, Columbia University Medical Center, New York, NY, USA; ^5^ The Taub Institute for Research on Alzheimer's Disease and The Aging Brain, Columbia University, New York, NY, USA; ^6^ Rush Alzheimer's Disease Center, Chicago, IL, USA; ^7^ Taub Institute for Research on Alzheimer's Disease and the Aging Brain, Columbia University, New York, NY, USA

## Abstract

**Background:**

Alzheimer's Disease (AD) subtyping using unsupervised clustering of omics data often results in subtypes with limited clinical relevance. To address this, we developed a novel supervised clustering method integrating longitudinal clinical data with high‐dimensional omics data, capturing the age‐dependent effects of AD risk factors such as ApoE4. This method enables the identification of clinically meaningful AD subtypes that better reflect disease heterogeneity.

**Method:**

We applied our method to 1,020 adults from the Religious Orders Study and Memory and Aging Project (ROS/MAP), incorporating longitudinal global cognition trajectories and differentially expressed genes from the prefrontal cortex in AD. Analyses were adjusted for sex, ApoE4, and vascular risk factors. We examined time‐varying effects of covariates on cognition within each subtype, assessed clinico‐pathological differences, and conducted survival analysis to estimate the time to AD dementia onset. Differential gene expressions and pathway analyses were performed to uncover molecular mechanisms associated with each subtype.

**Result:**

We identified four AD subtypes (Table 1), with cognitive decline following a gradient from Subtype 1 (slowest) to Subtype 4 (fastest) between ages 70‐100 (Figure 1a). In Subtype 3, women (ages 75‐85) and participants with stroke (ages 65‐100) experienced a decline in cognition. In Subtype 4, ApoE4 carriers had lower cognition after age 75, while diabetes (ages 77‐88) and frailty (before 75, after 93) were associated with cognitive decline (Figure 1b). Subtype 4 had the highest prevalence of AD dementia, beta‐amyloid load, tau pathology, and vascular pathologies (e.g., cerebral amyloid angiopathy, arteriolosclerosis, hippocampal sclerosis, Lewy bodies) and exhibited the steepest cognitive decline (Figure 2a) compared to Subtype 1. Survival analysis showed increasing AD dementia risk across subtypes. At age 85, the probability of developing AD dementia was 14.6% for Subtype 1, rising to 52.2%, 52.9%, and 77.0% for Subtypes 2–4, respectively (Figure 2b). Differentially expressed genes in Subtype 4 vs. Subtype 1 were enriched in pathways related to neural function, gene and protein regulation, and circadian rhythm.

**Conclusion:**

Our integrative approach identified clinically meaningful AD subtypes with distinct cognitive trajectories, vascular profiles, and molecular signatures. These findings provide critical insights for precision medicine and targeted interventions in AD.